# A split nozzle and two-protein chain-like fibers define the tail architecture of *Pseudomonas aeruginosa* podophage A1002

**DOI:** 10.1371/journal.pbio.3004081

**Published:** 2026-11-03

**Authors:** Hao Pang, Yuan Chen, Zeng Peng, Hao Xiao, Wenyuan Chen, Xiaowu Li, Ping Chen, Jing Zheng, Hongrong Liu

**Affiliations:** 1 Institute of Interdisciplinary Studies, Key Laboratory for Matter Microstructure and Function of Hunan Province, Key Laboratory of Low-dimensional Quantum Structures and Quantum Control, School of Physics and Electronics, Hunan Normal University, Changsha, China; 2 Department of Physics, Shaoyang University, Shaoyang, China; 3 GMU-GIBH Joint School of Life Sciences, The Guangdong-Hong Kong-Macao Joint Laboratory for Cell Fate Regulation and Diseases, Guangzhou Medical University, Guangzhou, China; 4 Department of Gastroenterology, The Second Affiliated Hospital, Guangzhou Medical University, Guangzhou, China; 5 College of Elementary Education, Changsha Normal University, Changsha, China; University of Glasgow, UNITED KINGDOM OF GREAT BRITAIN AND NORTHERN IRELAND

## Abstract

Phage therapy offers unique advantages against antibiotic-resistant bacteria. However, high-resolution structural studies of phages targeting *Pseudomonas aeruginosa*—a top-seven global pathogen—particularly podophages, remain severely limited, directly impeding structure-based development of novel antimicrobials. In this study, we utilize cryo-EM to resolve the high-resolution intact structure of mature phage A1002, a podophage infecting carbapenem-resistant *P. aeruginosa*. Based on density maps combined with AlphaFold3 predictions, we identified 11 structural protein components from the head to the tail. Phage A1002 exhibits a distinctly elongated tail morphology distinct from other reported podophages. Notably, the A1002 nozzle employs a distinct assembly strategy, featuring the unique nozzle-rope protein and nozzle-base protein, which may have evolved through modular evolution and horizontal gene transfer. Furthermore, the nozzle-rope protein provides 11 adsorption sites for fiber dimers, and these dimers are assembled from two proteins, giving rise to two types of dimers: dimers 1–7 correspond to fiber I (gp40), and dimers 8–11 correspond to fiber II (gp41). The nozzle-rope protein and these dimers combine to form a chain-like fiber. The tail tube comprises a double-layered, elongated tube structure that functions to simultaneously increase tail length and provide docking sites for six chain-like fibers. These distinctive structural features provide the molecular basis for A1002’s broad-spectrum infection of drug-resistant *P. aeruginosa* and offer a novel molecular blueprint for rational design of structure-guided phage therapeutics.

## Abstract 2

Drug-resistant Pseudomonas aeruginosa is difficult to treat, and its phages remain poorly understood. This study shows that the podophage A1002 has an unusual tail architecture that offers a blueprint for structure-guided antimicrobial design.

## Introduction

*Pseudomonas aeruginosa* is a highly environmentally adaptable gram-negative opportunistic pathogen and a primary causative agent of infections on a global scale. It can cause various severe diseases, including bloodstream infections, pneumonia, urinary tract infections, and wound infections, accompanied by persistently high mortality rates [1–4]. Its potent pathogenicity and resistance mainly stem from three intrinsic mechanisms: a naturally low-permeability outer membrane, an efficient efflux pump system, and a strong biofilm formation capability. Collectively, these mechanisms form an intrinsic resistance barrier against most antibiotics [5–7]. Furthermore, this bacterium has been shown to survive for extended periods in water, medical equipment, and even environments with disinfectant residues, thus serving as a significant cause of nosocomial infection outbreaks [8]. More alarmingly, this bacterium can rapidly acquire new resistance genes via gene mutation or horizontal gene transfer, thereby evolving into multidrug-resistant (MDR), extensively drug-resistant (XDR), or even pandrug-resistant (PDR) strains [9]. Consequently, the World Health Organization (WHO) has designated it as a top-priority “ESKAPE” pathogen, underscoring the urgent need for the development of novel antimicrobial strategies against it [10].

In the face of the severe challenge posed by traditional antibiotic resistance, phage therapy, with its advantage of specifically lysing bacteria without affecting human cells, has attracted widespread attention as an alternative or complementary strategy [11]. Especially in combating refractory infections caused by *P. aeruginosa* involving complex biofilms, such as lung infections in cystic fibrosis patients [5], phage therapy has shown unique potential [12]. Nevertheless, the primary impediment to the transition of this field from experimental exploration to precise clinical application is the significant absence of comprehension regarding the molecular mechanisms of phage infection and DNA ejection, particularly their high-resolution 3D structures. Presently, the structures of only a small number of *P. aeruginosa* phages have been resolved, including DEV [13], E217 [14], and Pa193 [15]. With regard to the structural studies of podophages specifically targeting drug-resistant strains, particularly clinical isolates, only DEV has been characterized, which is able to clear *P. aeruginosa* infections in both wax moth larvae and vertebrate models [13]. The absence of critical structural information impedes our comprehension at the atomic level of how phages recognize their hosts, adsorb, and penetrate the multiple outer membrane barriers (such as capsules and biofilms) on which *P. aeruginosa* relies for survival, and ultimately infect bacteria efficiently.

Among the numerous tailed phages, podophages are considered one of the most promising candidates for phage therapy owing to their relatively simple tail and broad distribution in natural and clinical environments. Podophages manifest divergent structural morphologies when infecting disparate types of bacteria, namely gram-negative and gram-positive. At present, the most representative structural studies on podophages infecting gram-negative bacteria, include T7-like [16–18] and P22-like [19–21] phages. These phages usually possess conserved tail components composed of a portal, adaptor, nozzle, and needle, and rely on lateral fibers for host recognition [13]. In contrast, the most typical structural studies of podophages infecting gram-positive bacteria include P68-like phages [22–24], which usually possess elongated tail components formed by the portal, stem, and tail needle, with fibers attached laterally. Notably, among podophages infecting gram-positive bacteria, P68-like elongated tails are extremely rare, having been reported only in a few phages such as N4-like [13,25,26] and human gut crassvirus [27–29]. This structural divergence may be indicative of the evolution of distinct tail strategies in podophages in response to differences in the cell membranes of gram-negative and gram-positive bacteria, particularly variations in cell wall thickness. It is evident that current reports on podophages tail diversity remain limited, and the structural understanding in this field is still based on a small number of typical model systems. Consequently, the systematic discovery and determination of the high-resolution structures of novel podophages from significant resistant pathogens, such as *P. aeruginosa*, is imperative to overcome current limitations and expand our understanding of podophage structural diversity. Concurrently, these studies will establish a fundamental structural framework for the rational design, engineering modification, and eventual clinical application of phages against drug-resistant bacteria.

Phage A1002 was isolated from sewage and demonstrated lytic activity against carbapenem-resistant *P. aeruginosa* (CRPA) strains [30]. The phage exhibited extensive lytic activity against the tested bacterial panel, with a plaque-forming rate of 76.36% (42/55). Genomic analysis indicated that A1002 belongs to the genus *Kochitakasuvirus*, which currently includes only two confirmed members, KPP25 [31] and R18 [32]. However, no structural data have been reported for either phage A1002 contains a 63.77 kb circular double-stranded DNA genome encoding 88 predicted open reading frames. Notably, no virulence factors or antibiotic resistance genes were identified in the A1002 genome. The results of the functional assays demonstrated that A1002 was capable of effectively inhibiting the growth of CRPA within 12 hours and achieving approximately 50% biofilm clearance. Furthermore, combination treatment with allicin significantly enhanced its antibacterial efficacy, suggesting that A1002 is a promising candidate for antimicrobial therapy [30].

In this study, we examined the two-dimensional structure of A1002 using electron microscopy, which revealed that A1002 possesses an icosahedral head and a short, non-contractile tail. However, its tail is distinctly longer and more complex than that of typical podophages. In order to further elucidate the architecture, we utilized cryo-EM to resolve the complete 3D structure of mature A1002 to a resolution where de novo models can be traced. Based on the density map, in conjunction with AlphaFold3 [33], we identified a total of 11 structural proteins, including the major capsid protein (MCP), cement protein (CP), portal protein, adaptor protein, collar protein, nozzle-rope protein, nozzle-base protein, tail tube protein, fiber dimer I, fiber dimer II, and tail needle. Notably, A1002 displays a distinct assembly strategy: the nozzle-rope protein and nozzle-base protein collectively execute the function of a typical nozzle protein. Furthermore, the nozzle-rope protein also provides adsorption sites for 11 fiber dimers, which collectively form a chain-like structure that winds tightly around the exterior of the elongated tail tube. It is hypothesized that this distinctive tail structure may confer advantages to A1002 when infecting *P. aeruginosa*, particularly in penetrating its complex extracellular barriers, such as capsules or biofilms. The high-resolution structure of A1002 establishes a representative structural model for phages of the genus *Kochitakasuvirus* and provides a structurally informed candidate for the future development of phage-based therapeutics against multidrug-resistant *P. aeruginosa* infections.

## Result

### Overall structures of *P. aeruginosa* phage A1002

Mature A1002 particles were purified from *P. aeruginosa* for cryo-EM data collection (S1A Fig). A total of 38,376 particles were extracted from 7,714 cryo-EM micrographs. Using RELION-3.14 software [34], we obtained an icosahedral structure of the A1002 head at a resolution of 3.4 Å (S1B Fig). The orientation parameters from the icosahedral reconstruction were converted into cryoSPARC [35], with the local structure of the head penton being reconstructed to 2.7 Å resolution (box size 400 × 400) and 3.2 Å resolution (box size 600 × 600), respectively (S1B Fig). In consideration of the complexity and extended length of the portal-tail complex, we focused on optimizing the orientation and center parameters of the portal-tail complex. Using cryoSPARC [35], we resolved the structures of the portal-nozzle complex, collar, the entire tail complex, and chain-like fibers at the tail tip (local fiber) at resolutions of 3.1 Å, 3.1 Å, 3.3 Å and ~3.8 Å, respectively (S1B Fig and S1 Table). Resolutions were estimated using the Fourier shell correlation (FSC) = 0.143 criterion following the “gold standard” method [36], shown in S1 Fig. Local resolution maps of A1002 are presented in S1C Fig, and the workflow for cryo-EM data processing and structure determination is illustrated in S2 Fig. The high-resolution maps enabled us to build atomic models for most structural proteins of A1002 (S3 Fig), including MCP gp50, CP gp49, portal protein gp52, adaptor protein gp47, collar protein gp33, nozzle-rope protein gp39, nozzle-base protein gp42, tail tube protein gp37, tail fiber I protein gp40, and tail fiber Ⅱ protein gp41. The needle protein gp29 was identified with the assistance of AlphaFold3 [33] (S3 Fig). We additionally observed an unassigned density at the interface between the collar and adaptor proteins (Fig 1A), which is attributed to an extra structural component and tentatively named the “bonding protein”. However, this protein could not be resolved structurally, most likely due to its inherent flexibility.

**Fig 1:**
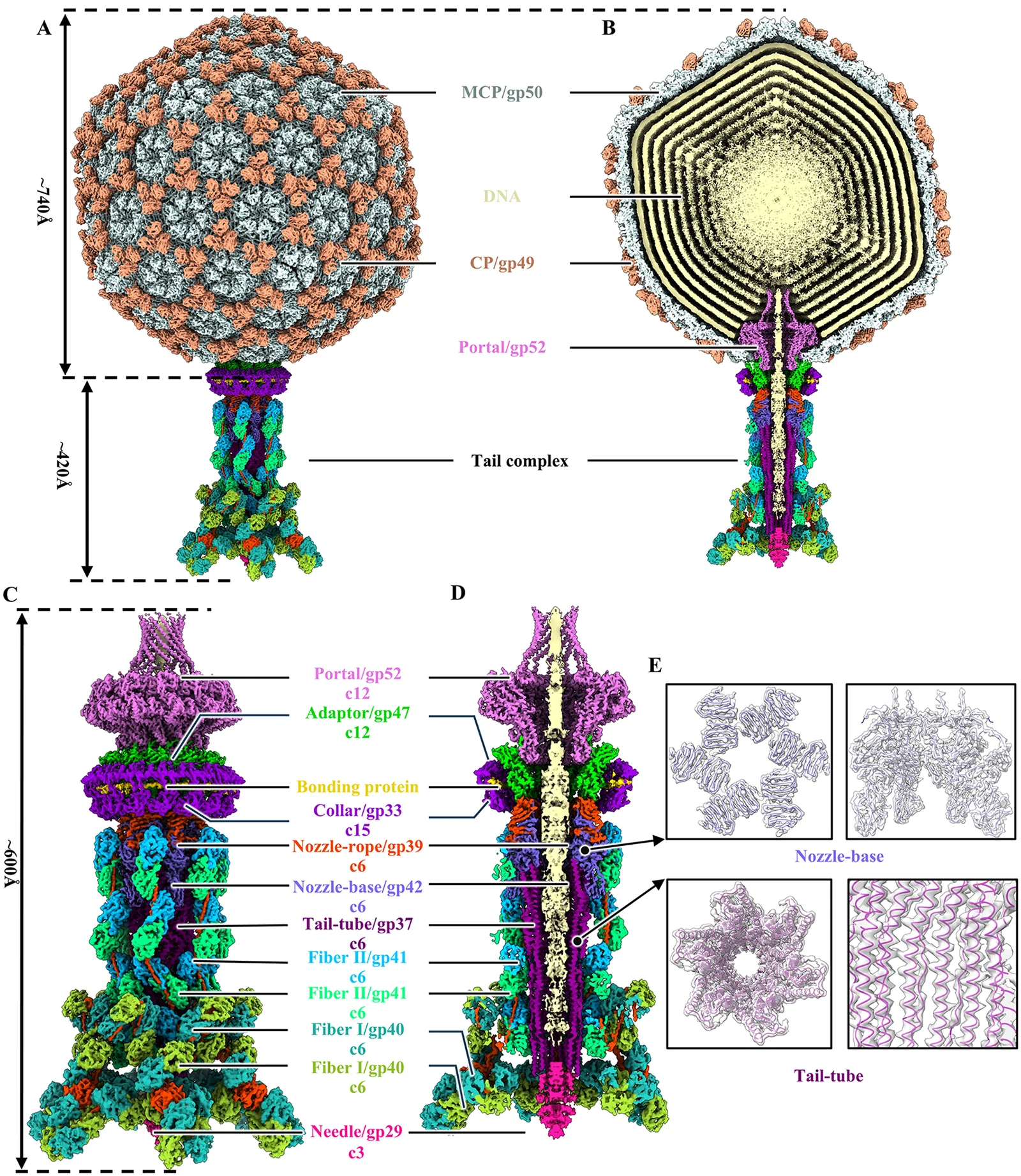
Overall structures of mature A1002. **(A and B)** Side (A) and cut-open (B) views of the asymmetric density map of mature A1002. **(C and D)** Side (C) and cut-open (D) views of the high-resolution density map of the portal-tail complex of mature A1002. **(E)** Superposition of the backbone models onto the top‑view (upper left) and side‑view (upper right) density maps extracted from the nozzle-base gp42 (PDB ID: 26FB), and onto the top‑view (lower left) and side‑view (lower right) density maps extracted from the tail tube gp37 (PDB ID: 44NX). The data underlying this Figure can be found in S1–S6 Data.

Mature A1002 particle possesses a head of approximately 70 nm in diameter and a tail of about 40 nm in length, with the tail attached to the head via the portal protein (Fig 1A). The A1002 capsid is composed of 535 copies of MCP gp50, organized into an icosahedral shell with a triangulation number of 9 (Fig 1A). 175 copies of the trimeric CP gp49 are attached at the 3-fold axes and quasi-3-fold axes on the capsid surface, thereby further enhancing capsid stability (Fig 1A). The distinctive portal-tail complex is organized into four structural modules (Fig 1B–1D). The first module comprises a dodecameric portal, a dodecameric adaptor, a bonding protein, and a 15-fold symmetric collar. The second module comprises a hexameric nozzle-rope and a hexameric nozzle-base (Fig 1E). The third module comprises a dodecameric tail tube and a trimeric needle (Fig 1E). The fourth module is characterized by the presence of six chain-like fibers, which tightly wrap around the entire elongated tail tube. Each chain-like fiber consists of 11 fiber dimers that attach in a tandem manner to the N-terminus of the nozzle-rope protein (Fig 1C and 1D).

### Structure of A1002 head

Based on our cryo-EM map, we built the atomic model of the gp50 monomer from residues 3–375 (out of the 376 residues) (S3 Fig). The MCP gp50 of A1002 belongs to the classic HK97-like fold [37] and can be divided into five domains (Fig 2A): N-arm (residues 3–51), E-loop (residues 52–98), P-domain (residues 99–146 and 319–367), G-loop (residues 147–180), and A-domain (residues 181–318 and 368–375). Comparison with the MCPs of phages E217 [14], T4 [38], R4C [39], and Pa193 [15] indicates that the A1002 MCP adopts a conserved architecture, featuring a flexible N-terminal loop followed by a globular domain that extends into a β-strand-rich domain (S4A Fig). The superimposition of the nine gp50 monomers in an asymmetric unit reveals obvious conformational differences in the N-arms and E-loops (S4B Fig). These structural changes enable the formation of two distinct types of capsomeres, including 11 pentons and 80 hexons. Notably, the A-domain of monomers in pentons adopts a distinct conformation compared to that in hexons, resulting in different curvatures between the two capsomere types that are necessary to accommodate the icosahedral geometry (S4C Fig).

**Fig 2:**
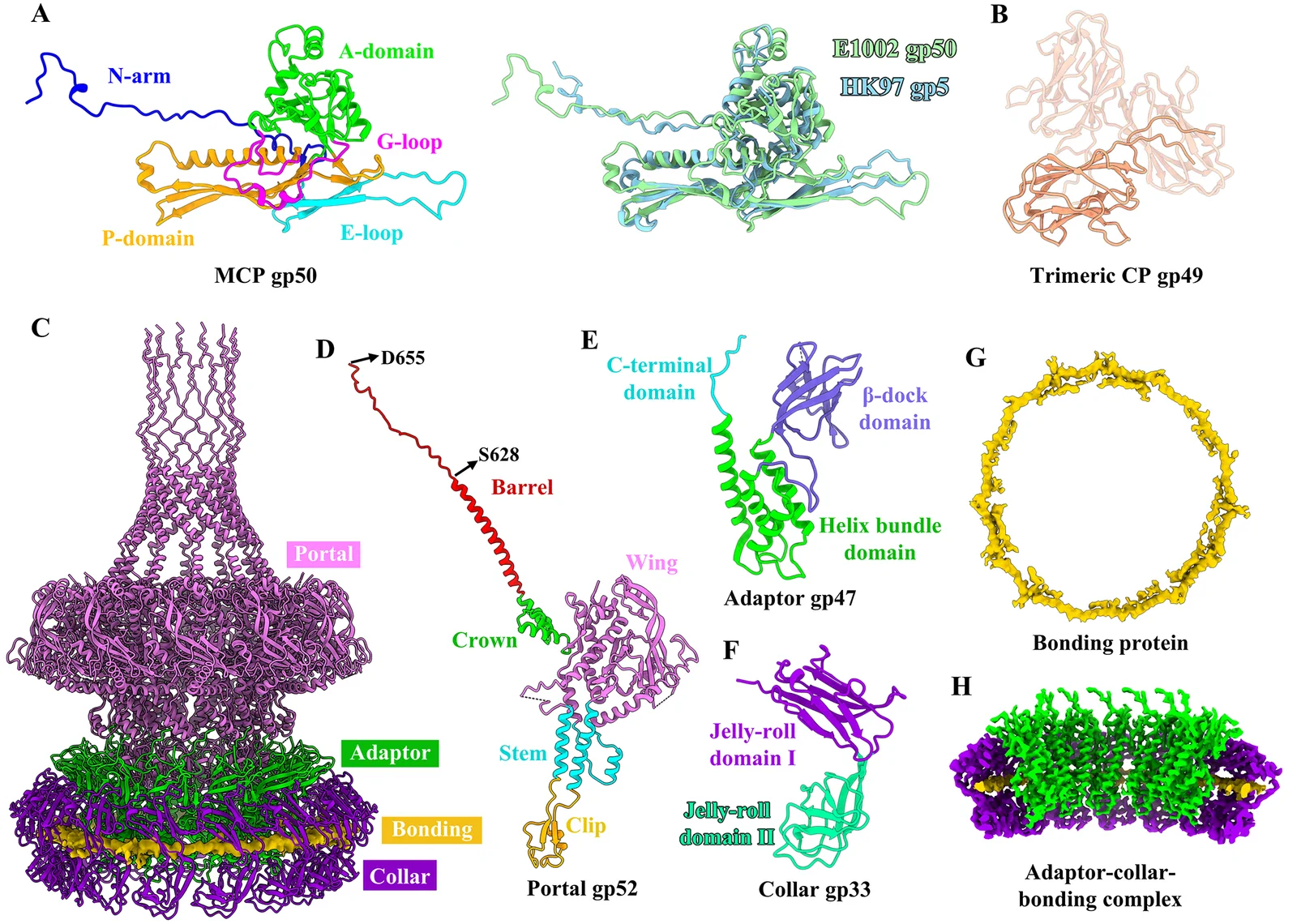
Structures of the head, portal-adaptor complex of A1002. **(A)** Left: Ribbon model of the MCP gp50 shown in five domains (PDB ID: 44NY). Right: Structural comparison between the A1002 MCP and the HK97 MCP (PDB ID: 1OHG). **(B)** Ribbon model of the trimeric CP gp49 (PDB ID: 44NY). **(C)** Side view of the portal-adaptor complex composed of the portal, adaptor, bonding protein and collar (PDB ID: 26FB/46KE). **(D–F)** Ribbon models of the portal protein gp52 **(D)**, the adaptor protein gp47 **(E)**, and the collar protein gp33 **(F)**. All the proteins are colored according to their domains. **(G)** Top view of the density map of the bonding protein (EMDB ID: 83847). The data underlying this Figure can be found in S4 Data. **(H)** Cross-section of the interaction among the collar, adaptor, and bonding (EMDB ID: 83847), the data underlying this Figure can be found in S4 Data.

At the 3-fold and quasi-3-fold axes of the icosahedral capsid, 175 copies of the trimeric CP gp49 decorate the capsid surface (Figs 1A and 2B). We traced 140 of the total 143 residues (residues 4–143) for each gp49 monomer (S3 Fig). Structurally, CP gp49 adopts a classic jelly roll motif consisting of two antiparallel β-sheets (S3 Fig) [40]. Trimeric gp49 exhibits structural and functional similarity to the cement proteins gp15 in phage KHP30 [41], gp6 in phage Pam1 [42], and gp1 in phage GP4 [43,44] (S5 Fig). These proteins are typically situated on the outer surface at the penton–hexon and hexon–hexon interfaces, thereby reinforcing capsid stability. Notably, multiple salt bridge interactions are observed between the inter-capsomer (S6A–S6C Fig), including K60–D114 and D100–R116, where the first residue of each pair belongs to one capsomer and the second to the neighboring capsomer (S6D Fig). In addition to these interactions, the trimeric CP gp49 forms extensive hydrogen bonds with MCPs from different capsomers at the 3-fold and quasi-3-fold axes of the A1002 capsid, further reinforcing capsid stability. Specifically, the residue T136 of CP gp49 forms a hydrogen bond with the residue K339 of MCP gp50, and the same residue T136 also pairs with N81 of MCP gp50 (S6E Fig).

### Structure of portal-adaptor-collar-bonding complex in A1002

The A1002 portal consists of 12 copies of gp52, which are structurally conserved across reported portal proteins from Pam1 [42], P-SCSP1u [17], DEV [13] and N4 [25] (S7 Fig). Following the protein nomenclature, each gp52 can be divided into five structural regions (Fig 2C and 2D): the wing domain (residues 8–55, 80–209, 247–347 and 440–541), the clip domain (residues 371–421), the stem domain (residues 56–79, 348–370 and 422–439), the crown domain (residues 542–590), and the C-terminal barrel domain (residues 591–655). The 12 copies of the barrel domain of the portal assemble into an elongated cylindrical barrel structure that extends into the capsid interior. Nearly a hundred residues (residues 628–730) of the barrel domain could not be resolved in the density map, indicating the inherent flexibility of this region (Fig 2C). This feature is reminiscent of the portals observed in phages GP4 [43], VP1 [45], and P22 [46], in which the portal typically interacts with the internal core proteins. The adaptor consists of 12 copies of gp47, which exhibits significant structural similarity to adaptors in other tailed phages, such as SU10 [47], Sf6 [19] and Carin-1 [18] (S8 Fig). Each gp47 comprises three domains (Fig 2E): a helix bundle domain (residues 2–48 and 158–208), a β-dock domain (residues 49–157), and a C-terminal domain (residues 209–219). In the majority of gram-negative podophages, the β-dock domain of the adaptor fulfils a conserved role in connecting 6- or 12-fold symmetric tail fibers to the tail, such as T7-like [48,49] and P22-like phages [50,51]. Conversely, in phage A1002, the β-dock domain of the 12-fold symmetric adaptor functions as a scaffold for a 15-fold symmetric collar protein, gp33, thereby forming a distinctive 12:15 symmetry-mismatched assembly (Fig 2C). Furthermore, a central bonding protein mediates the adaptor-collar connection. However, the precise symmetry of this protein remains undetermined owing to its inherent flexibility (Fig 2C). To date, the phenomenon of additional accessory collar proteins decorating the adaptor periphery has been reported in only a few podophages, namely phages DEV [13] and GP4 [43]. In DEV and GP4, these accessory proteins provide additional attachment sites for tail fibers. In A1002, however, this 12:15 symmetry-mismatched assembly may instead serve to reinforce the mechanical stability of the adaptor. This is significant because the adaptor is the first key protein linking the portal to the elaborate tail component, and must support its complex tail architecture.

Based on the density map, we built the atomic model of the collar protein gp33 encompassing residues 5–195 out of a total of 197 amino acids (S3 Fig). Protein gp33 adopts a double jelly-roll fold architecture comprising jelly-roll fold domain I (residues 5–21 and 110–195) and jelly-roll fold domain II (residues 22–109) (Fig 2F). This structural arrangement is analogous to the jelly-roll fold observed in gp13 of myophage Milano [52], and the single jelly-roll fold domain in gp33 is analogous to the collar protein attached to the adaptors in DEV [13] and GP4 [43] (S9A Fig). The interaction between the adaptor and the collar protein is primarily mediated by electrostatic complementarity, with the β-dock domain of the adaptor and the jelly-roll fold domain I of the collar forming the basis of this interaction (S9B Fig). Furthermore, the bonding protein is tightly intercalated at the interface between the interacting domains of these two proteins, further augmenting the stability of the adaptor-collar complex (Figs 2G and 2H). However, due to inherent flexibility, the precise atomic model and symmetry of the bonding protein remain undetermined (Fig 2G). A similar phenomenon, in which only a small portion of the ring-shaped accessory protein attached to the adaptor could be resolved, has also been observed in phage VP1 [45], suggesting that the remaining regions of these proteins are too flexible to be visualized in cryo-EM density maps.

### The nozzle-rope and nozzle-base proteins in A1002

The A1002 adaptor is attached to a unique hexameric nozzle-rope protein gp39, which subsequently connects to the hexameric nozzle-base protein gp42 (Fig 3A). The combination of gp39 and gp42 exhibits a distinctive division of labor, akin to a heterodimeric nozzle, thus to accommodate the complexity and high flexibility of its tail region. Due to the structural intricacy of the A1002 tail, we integrated two reconstruction maps, encompassing the entire tail complex, and chain-like fibers at the tail tip, to ascertain the complete density of gp39. On this basis, we built the atomic model of the protein gp39 from residues 3–32 and 96–620, out of a total of 620 amino acids (S3 Fig). For the remaining regions, due to their flexibility, it was only possible to generate a backbone model using ModelAngelo software [53]. According to the interactions of gp39 with other tail proteins, gp39 can be divided into four domains: the N-terminal platform-C domain (residues 520–620), the β-propeller-C domain (residues 469–519), the fiber-dock domain (residues 243–468, 3–32 and 96–107), and the C-terminal immunoglobulin-like (Ig-like) domain (residues 108–242) (Fig 3B). The platform-C domain is composed of a two-β-sheet fold, which is structurally similar to the platform domain of nozzle proteins in podophages [16,17,42] (S10 Fig). The β-propeller-C domain of gp39 exhibits a structural similarity to the typical β-propeller blade architecture of nozzle proteins, although it comprises only two incomplete blades. A structural similarity search using the DALI server [54,55] revealed that gp39 shares structural similarity with the nozzle proteins of phage VP1 [45] and phage Sf6 [19] (S10 Fig). This similarity can be attributed to the fact that the platform and β-propeller domains of gp39 maintain the core folds and functional characteristics of the corresponding domains in classical nozzle proteins. The fiber-dock domain contains a rope-like structure approximately 300 Å in length, which runs closely along the tail, bending downward and extending to the distal end of the tail, thereby providing binding sites for tail fiber dimers (described below) (Fig 1A). The Ig-like domain, which terminates the extension of the fiber-dock domain, contains two β-sheets (Fig 3B).

**Fig 3:**
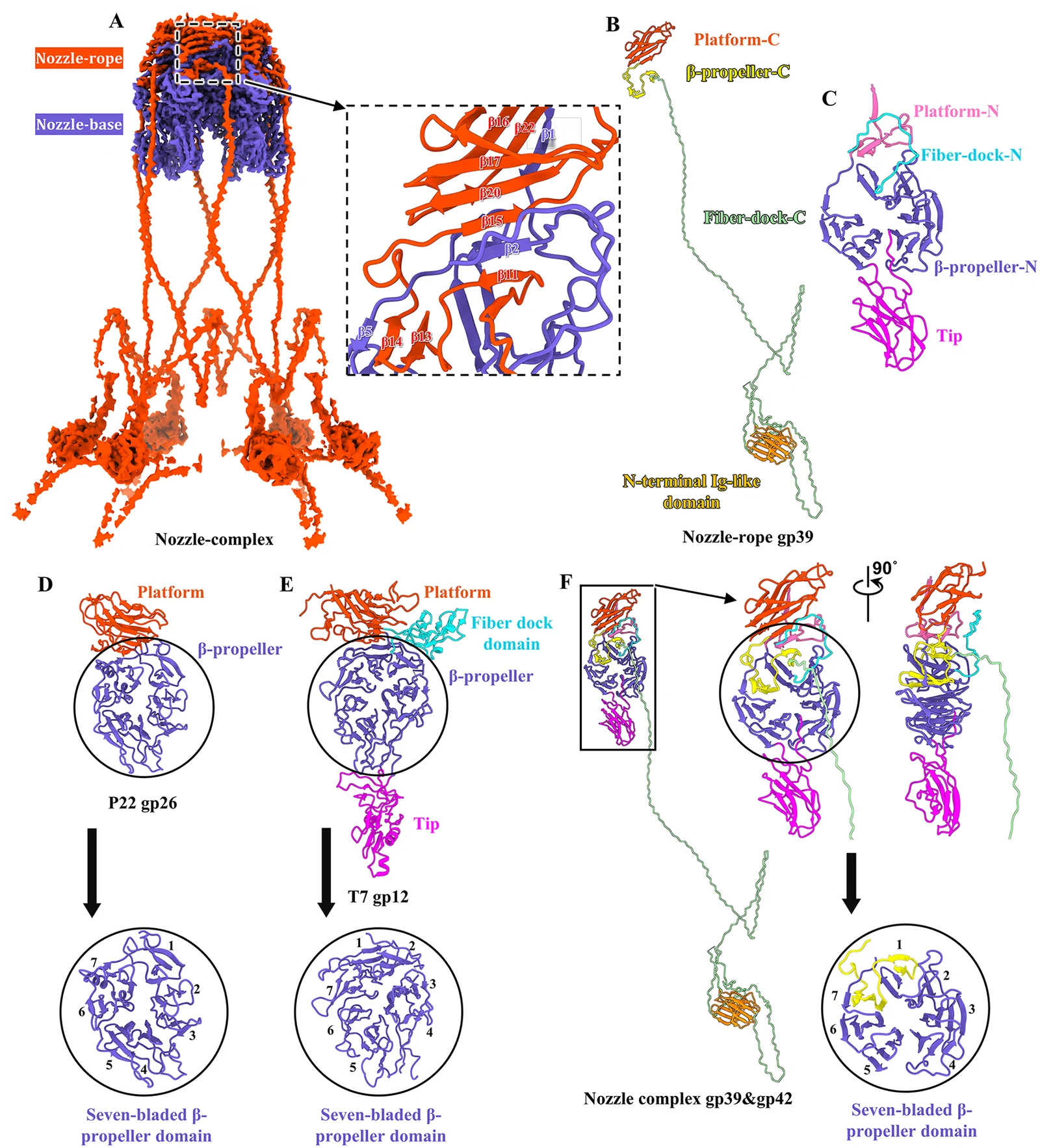
Structures of the nozzle-rope and nozzle-base of A1002. **(A)** Side view (left) and zoomed-in (right) views of the interactions of the nozzle-rope (orange-red) (PDB ID: 44NY/26GP) and nozzle-base (slate blue) (PDB ID: 26FB). **(B and C)** Ribbon models of the nozzle-rope protein gp39 (B) and nozzle-base protein gp42 (C). All the proteins are colored according to their domains. **(D and E)** Ribbon models of the nozzle gp26 (PDB ID:9JG6) in P22 and gp12 (PDB ID:7BOY) in T7. All proteins are colored according to their domains. The insets show the zoomed-in views of the seven-bladed β-propeller domain. **(F)** Zoomed-in views of the nozzle-like domain and seven-bladed β-propeller domain of the nozzle-rope and nozzle-base complex in A1002. The color coding is identical to that used in Fig 3B and 3C.

We also built the atomic model of the protein gp42 from residues 3 to 396 (out of 398 residues) (S3 Fig). Following the canonical nozzle nomenclature, gp42 comprises four domains (Fig 3C): the platform-N (residues 3–43), β-propeller-N (residues 44–123 and 232–373), fiber-dock-N (residues 374–396), and nozzle-tip domains (residues 124–231). Compared with the conserved nozzle proteins found in podophages T7 [56], Pam1 [42], VP1 [45], Carin-1 [18], and Sf6 [19] (S10 Fig), the nozzle-base protein gp42 of A1002 retains the core framework but exhibits pronounced truncations in key domains (Fig 3C). The platform-N domain is reduced to only two β-strands and a connecting loop. The nozzle β-propeller domain in podophages generally contains the complete nine-bladed β-propeller domain (Figs 3D and 3E). However, in the β-propeller-N domain of A1002 gp42, blade 1 lacks two β-strands, while blade 2 lacks three (Fig 3F). The C-terminal region of fiber-dock-N is also truncated (Fig 3C). To compensate for these structural deficiencies, the platform-C and β-propeller-C domains of the nozzle-rope protein gp39 fill in the portions missing from the platform-N and β-propeller-N domains of the nozzle-base protein gp42, respectively (Fig 3F), by forming enhanced β-strands (Fig 3A). Through this structural complementation, the nozzle-rope protein and the nozzle-base protein together reconstitute the architecture and function of a canonical nozzle protein.

### The tail-tube protein gp37 and tail needle gp29 in A1002

Based on our cryo-EM density map, we traced 302 amino acids from residues 4–305 of the total 318 residues of the tube protein gp37 (S3 Fig). Based on the interaction environment of the fiber and nozzle-base protein with the tube, we divided the tube gp37 into three structural domains (Fig 4A and 4B): the peripheral fiber-dock domain (residues 4–105), the nozzle-dock domain (residues 106–186), and the inner channel domain (residues 187–305). The peripheral fiber-dock domain is primarily composed of three α-helices, which are connected by multiple loops, forming an irregularly extended structure, whereas the inner channel domain is composed of two helices, forming a regularly extended helical structure (Fig 4B). The nozzle-dock domain is constituted by two antiparallel helices (Fig 4B). Twelve gp37 monomers assemble into a complex tail tube structure with a height of approximately 240 Å (Fig 4C–4F). The 12 peripheral fiber-dock domains form an irregular 12-fold symmetric outer wall of the tail tube with an outer diameter of approximately 60 Å (Fig 4F). The 12 inner channel domains are arranged in 12-fold symmetric left-handed spirals, forming a regularly extended channel with an inner diameter of approximately 40 Å (Fig 4E). The different conformations of the nozzle-dock domains of the two adjacent gp37 monomers result in the 12 nozzle-dock domains being arranged in pseudo-6-fold symmetry (Fig 4B). In this arrangement, the helices from every two monomers are positioned in close proximity to each other, collectively constituting a four-helix bundle structural unit (Fig 4D). This transition from pseudo-6-fold symmetry at the upper terminus of the tail tube (which serves to coordinate the 6-fold symmetric nozzle complex) to regular 12-fold symmetry throughout the main body of the tail tube represents the unique assembly architecture of the A1002 tail tube. Notably, the double-layered tail tube of A1002, which is characterized by an irregular outer wall and a regular inner channel, is distinctly different from those of other known podophages. In particular, the tail tubes of N4-like phage family members, such as N4 [25], DEV [13], Moo19, and B2 [26] adopt single-layered, α-helix-based 12-fold symmetric structures composed of α-helices (S11A Fig). In contrast, several gram-positive podophages, such as P68 [22], Andhra [24], and phi29 [23], possess 12-fold symmetric tail proteins with single-layered, funnel-shaped structures, whose tubular portions typically form 24 antiparallel β-strands arranged in right-handed spirals (S11A Fig). The distinctive double-layered structure of A1002 may result in the expression of unique functions: the robust inner tail tube exhibits prominent negatively charged residues, which may facilitate DNA release (S11B Fig), analogous to the tail tubes of siphophages and myophages [39,57,58]. The outer tail tube structure may provide an adaptive interface for the attachment and conformational changes of tail fibers (S11B Fig), thereby better accommodating the complexity of the host cell surface during infection.

**Fig 4:**
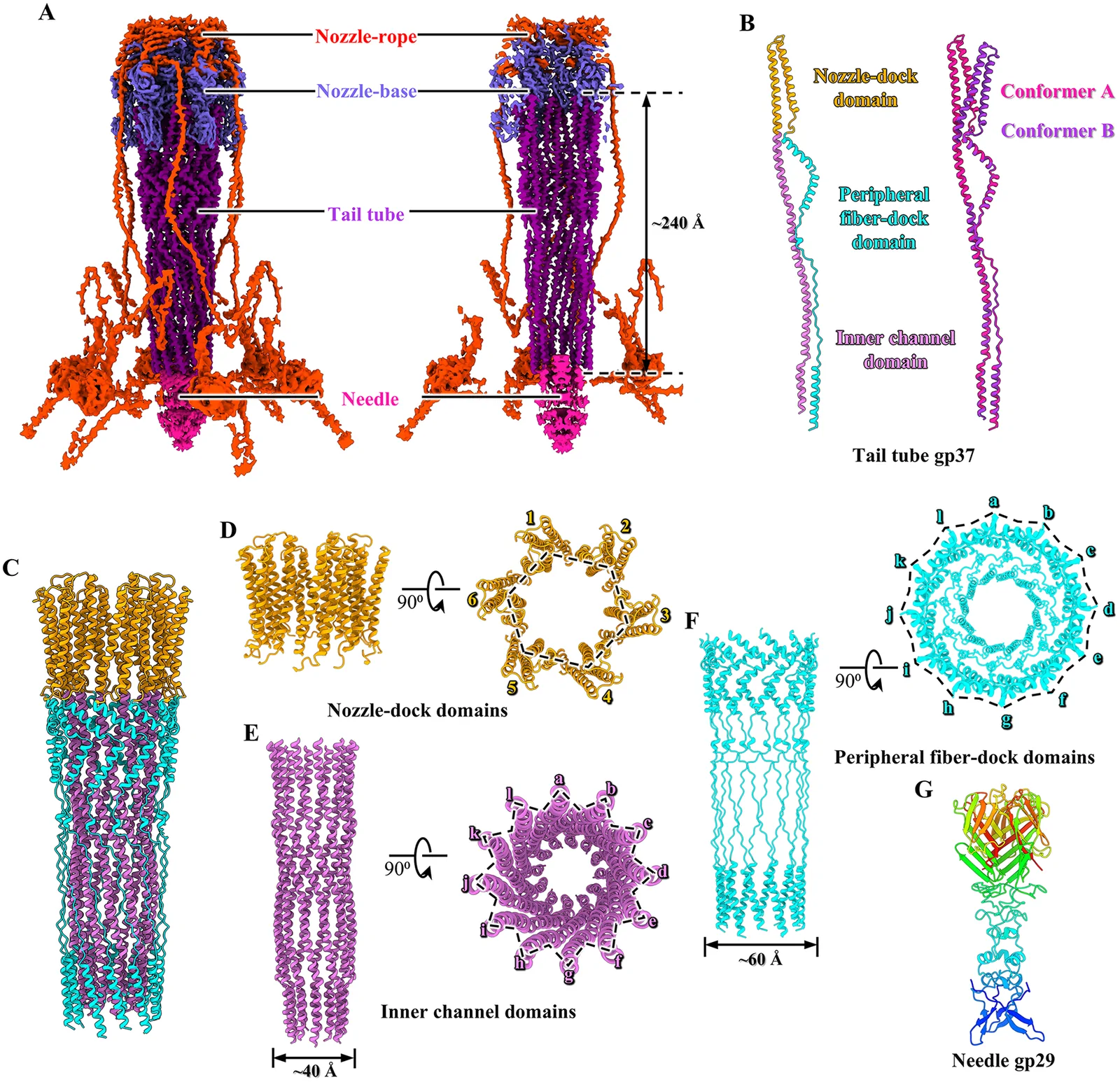
Structures of the tail tube and needle of A1002. **(A)** Side (left) and cut-open (right) views of the high-resolution density map of the tail complex from the nozzle-rope to the needle (EMDB ID: 82741/80643). The data underlying this Figure can be found in S5 and S6 Data. **(B)** Left: Ribbon model of the tail tube protein gp37 colored according to its domain. Right: Structural comparison between two conformations of gp37 (PDB ID: 44NX). **(C)** Side view of the 6- to 12-fold asymmetric tail tube. **(D, E, and F)** Side and top views of 3 structural domains of the tail tube with 6-fold (D) and 12-fold (E and F) symmetry. **(G)** Ribbon model of the trimeric needle protein gp29 modeled by AlphaFold3 and shown in rainbow colors.

The distal extremity of the tail tube is sealed by a tail needle (Fig 4A). Despite the implementation of various reconstruction strategies on the distal tail region, high-resolution density for the tail needle could not be obtained. This challenge is consistent with observations in other tailed phages [59–62], where the high intrinsic flexibility of the distal tail needle has consistently precluded high-resolution structural determination, with most available structures having been solved by X-ray crystallography [63,64]. Among tailed phages, the tail needle is known to generally assemble as trimers and share conserved structural features, in which the distal structure is typically formed by stacked α-helices [65,66] or β-sheets [67–69]. In order to identify the tail needle protein of A1002, we used AlphaFold3 [33] to predict the trimeric conformations of all 88 open reading frames with the exclusion of those encoding confirmed structural proteins. The results revealed that the predicted trimeric model of gp29 exhibited the characteristic morphology of a tail needle (Fig 4G). This finding led to the proposal of gp29 as the most probable candidate for the tail needle. Further structural similarity searches using Dali/TM-align [54,55,70] showed that gp29 shares significant structural homology with the tail fiber receptor-binding protein gpJ in Lambda [71], gp8 in *Lactobacillus* phage TP901−1 [72], gp6 in *Lactobacillus* phage P2 [73], and the tail receptor-binding proteins of *P. aeruginosa* R1- and R2-type pyocins [74] (S12 Fig). The N-terminus of all these proteins contains a conserved Ig-like fold domain, while their distal structures exhibit considerable diversity, consisting primarily of α-helices or β-sheet layers stacked in distinct arrangements (S12 Fig). This variation in distal architecture likely reflects specific evolutionary adaptations for recognizing different host surface receptors.

### The novel arrangement of chain-like fiber in A1002

As with the density acquisition of the nozzle-rope protein gp39, the complete density of the entire elongated chain-like fiber was also obtained by integrating two reconstruction maps, including the entire tail complex and chain-like fibers at the tail tip. The tail fiber is composed of 11 fiber dimers, which are sequentially attached to the fiber-dock domain of the nozzle-rope protein gp39, thereby forming a functional chain-like fiber (Figs 1A and 5A). Further examination reveals that these dimers exhibit slight conformational differences and are composed of two different proteins, a feature reported for the first time in tailed phages. For clarity, the 11 fiber dimers were numbered sequentially from 1 to 11, starting from the distal tip of the tail (Fig 5A). Dimers 1–7, located proximally at the distal tip and exhibiting a “stacked” arrangement, are shorter and wider; we refer to this type as fiber dimer I, which is composed of gp40 protein (Figs 5B and 5C). In contrast, dimers 8–11, situated closer to the nozzle complex and adopting an “extended” conformation, are longer and slender; we refer to this type as fiber dimer II, composed of gp41 protein (Figs 5B and 5C). We traced 144 amino acids (residues 2–145) of the 147-residue gp40, and modeled 138 residues (residues 34–171) of the 171-residue gp41 (Figs 5B and 5C). Although gp40 and gp41 share relatively low sequence similarity (16%), they exhibit remarkably high structural similarity. Superposition of the two types revealed only minor conformational differences with Cα root mean square deviation value (RMSD) of 3.47 Å (Fig 5C). Further comparison shows that both gp40 and gp41 adopt a structure similar to that of the fiber monomer gp80 of phage GP4 [43] and fiber monomer gp11 of VP1 [45] (Fig 5D). Notably, the GP4 gp80 contains 140 amino acids, the VP1 gp11 contains 160 amino acids, the A1002 gp40 contains 147 amino acids, whereas gp41 is longer, with 171 amino acids. However, in our model, gp41 lacks approximately the first 30 residues. In the high-resolution map of the fiber dimer, which interacts with the fiber-dock domain of the nozzle-rope protein gp39, no corresponding density was observed for these 30 N-terminal residues in any gp41 monomer (S3 Fig), suggesting that these amino acids may have been removed by proteolytic cleavage. The association of two gp40/gp41 monomers to form a dimer is facilitated by hydrophobic interactions (Fig 5E and S2 Table), and this interaction pattern is analogous to that observed in the fiber dimers of GP4 [43] and VP1 [45]. Within the complete chain-like fiber, each of the 11 fiber dimers forms an augmented β-sheet fold with the fiber-dock domain of the nozzle-rope protein gp39 (Fig 5F).

**Fig 5:**
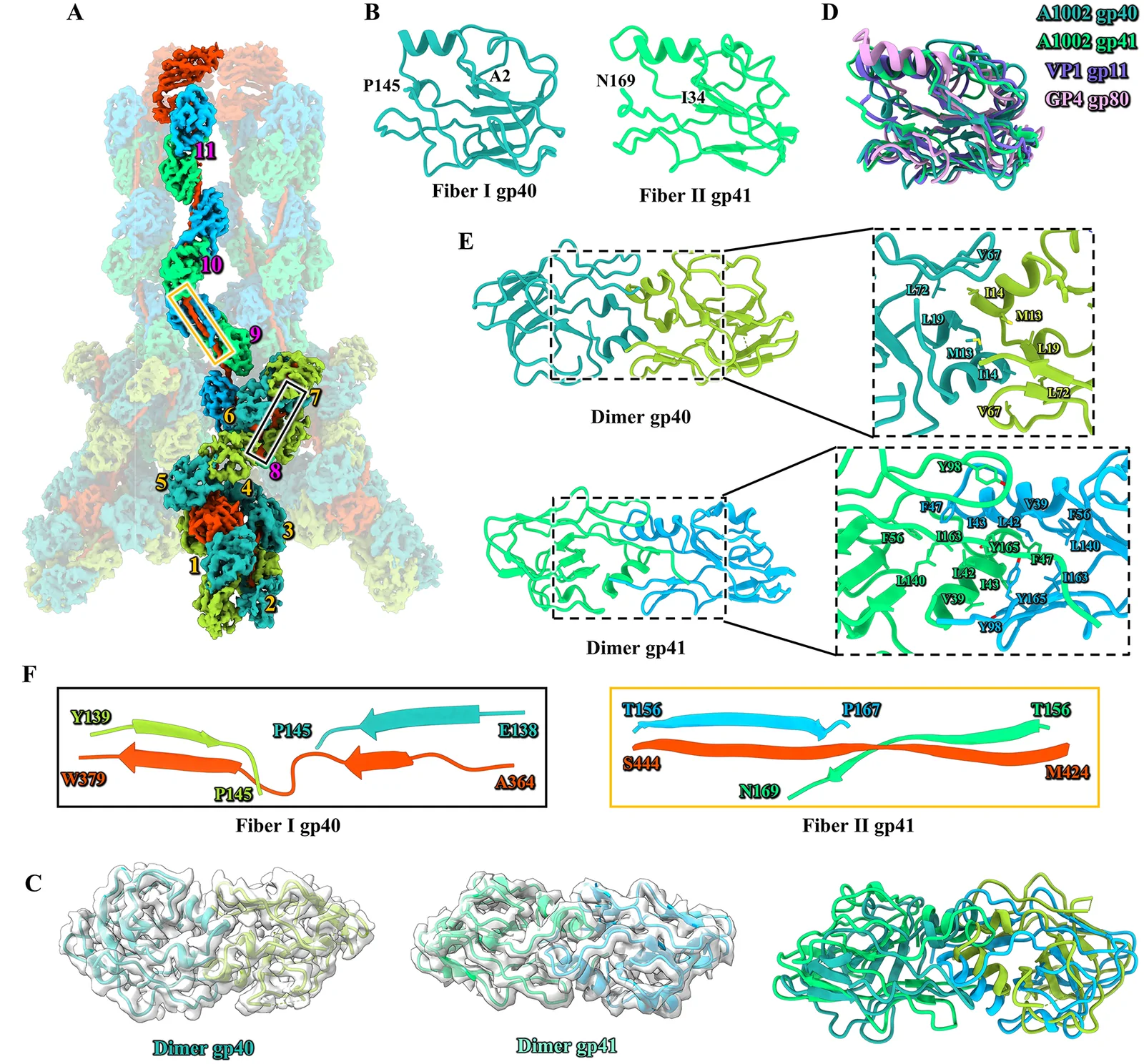
Structure of the chain-like fiber. **(A)** Side view of the high-resolution density map of the chain-like fibers (EMDB ID: 82741/80643). The data underlying this Figure can be found in S5 and S6 Data. The fiber-dock-C domain and the Ig-like domain of the nozzle-rope are shown in orange-red; each fiber dimer is colored alternately. Along the N-terminus to C-terminus direction of the nozzle-rope protein, 11 fiber dimers are numbered 1 to 11; among them, dimers 1–7 are shorter and wider (sky blue and spring green), designated as dimer I gp40, and dimers 8–11 are longer and more slender (yellow green and light sea green), designated as dimer II gp41. **(B)** Ribbon models of fiber I gp40 monomer (left) and fiber II gp41 monomer (right) (PDB ID: 44NX). **(C)** Structural comparison of ribbon models fitted into the density maps for fiber I gp40 dimer (left) and fiber II gp41 dimer (middle) (EMDB ID: 82,741) and superposition of the two types of the fiber dimers (right). The data underlying this Figure can be found in S5 Data. **(D)** Structural comparison of fiber dimeric monomers among A1002 gp40 and gp41, VP1, and GP4 (PDB ID: 8JOU). **(E)** Left: Ribbon models of fiber I gp40 dimer (top) and fiber II gp41 dimer (bottom). Right: Hydrophobic interactions between the two fiber monomers within a dimer. The hydrophobic side chains are labeled. **(F)** Zoomed-in view of the interactions between the fiber dimer and the fiber dock C domain of the nozzle-rope, extracted from Fig 5A.

The overall arrangement of the A1002 chain-like fiber is reminiscent of those of phages GP4 [43] and VP1 [45], in that all three are assembled from multiple fiber dimers. However, they differ substantially in their detailed organization. For individual dimers, both VP1 and GP4 exhibit only a single dimer conformation, whereas A1002 is composed of two different proteins forming two distinct dimers. In GP4, five dimers are connected by a separately encoded rope protein to form the chain-like fiber, which extends upward from the tail and docks onto the capsid in the mature state. In VP1, owing to the flexibility of the chain-like fiber, only three dimers could be resolved, with no additional protein identified as an anchoring element for the dimers. In the VP1 mature state, the fiber is similarly attached to the tail and extends upward. Notably, the A1002 chain-like fiber exhibits a significantly greater length compared to those observed in phages GP4 and VP1. The 11 fiber dimers of A1002 wrap around the fiber-dock domain of the nozzle-rope protein gp39 in a vine-like manner. The entire fiber extends from the nozzle along the base of the tail tube, displays a progressive morphological transition from the proximal extended region (dimers II 8–11) to the distal stacked region (dimers I 1–7) (Fig 5A). This structural architecture may confer a mode of motion distinct from those of GP4 and VP1: the proximal region may provide flexibility for target searching, whereas the distal region may function as a mechanosensitive switch, remaining folded in the resting state but, upon proper triggering, transmitting an infection signal to the phage through conformational changes. Meanwhile, the formation of two distinct dimers by two different proteins may enable A1002 to recognize a broader range of hosts during receptor binding. This possibility will be discussed further in the following section.

## Discussion

In this study, we resolved the intact structure of *P. aeruginosa* podophage A1002 by cryo-EM. Based on the high-resolution density map, we built atomic models for almost all proteins from the head to the tail in the mature particle. Comparing A1002 with typical podophages infecting gram-negative bacteria, such as T7-like and P22-like phages, as well as podophages infecting gram-positive bacteria, such as P68-like phages, reveals striking differences in its tail architecture. This suggests that A1002 may possess unique evolutionary advantages.

The A1002 is composed of a heterodimer-like nozzle formed by the nozzle-rope protein gp39 and the nozzle-base protein gp42. This is in contrast to the canonical architecture observed in most podophages infecting gram-negative bacteria, in which the nozzle is formed by a single protein containing four conserved domains: the platform domain, β-propeller domain, fiber-dock domain, and tip domain [16,19,75]. We propose that in A1002, the structure and function of the ancestral single nozzle protein became partitioned into a heterodimeric complex formed by gp39 and gp42. This process was likely driven by modular evolution and horizontal gene transfer. In particular, the genes encoding gp39 and gp42 may have remained in close proximity within the genome, potentially forming a functional “module”. This newly acquired genetic module, which may later have evolved into gp39, did not replace the ancestral nozzle gene; instead, it became functionally complementary to the modified or truncated ancestral nozzle gene, which may later have evolved into gp42. Over time, the two proteins gradually co-adapted in sequence and structure, thereby ultimately giving rise to the tightly associated heteromeric complex that is currently observed. The gp39-gp42 complex further appears to confer evolutionary advantages, with gp39 functioning as a “rope” that provides multiple docking sites for tail fiber dimers. This may result in a degree of spatial flexibility and a broader search for host range in comparison to traditional rigid tail protein components.

The chain-like fiber of A1002 is assembled from two different proteins that form two distinct dimers, a feature that has not been previously described in other chain-like fibers, including GP4 [43] and VP1 [45]. On this basis, we propose that the overall motion of the chain-like fiber results from the combined effects of rigid-body movement and flexible deformation. The distal region (dimers II 8–11), composed of longer and slender dimers, appears to be relatively stable and forms an extended rigid “linear” structure through attachment to the fiber-dock domain of the nozzle-rope protein gp39. This region may primarily undergo large and reversible scanning movements via rigid-body rotation, facilitating exploration of the host cell surface. In contrast, the proximal region (dimers I 1–7), in which the shorter and wider dimers are tightly stacked, is not completely rigid itself and may undergo conformational rearrangements upon specific signals, such as host receptor binding. It is even conceivable that a transition from dimers I to dimers II could be achieved, thereby facilitating the transmission of the binding signal through the fiber to the tail. However, such a transition may require the overcoming of a certain energy barrier, therefore, it would not occur spontaneously, thus ensuring the structural stability of the fiber in the absence of triggering signals. Furthermore, we propose that this chain-like fiber contains two equivalent dimer types, each composed of a distinct protein, and that both are likely capable of receptor recognition. This dual-component dimer design may facilitate phage A1002 to recognize a broader range of host receptors, thereby expanding its host range to a certain extent. The coordinated interaction between the 11 fiber dimers and the fiber-dock domain of gp39 may enable the tail fiber to probe host receptors with greater flexibility, across a wider spatial range and have a broader host range, potentially enhancing the infectivity of the phage toward diverse clinical isolates of *P. aeruginosa* and conferring a competitive ecological advantage in complex microbial communities.

In comparison to the typical podophages (such as T7-like and P22-like), A1002 exhibits a significantly more elongated tail. It has been previously reported that podophages with elongated tails generally fall into three distinct types. Firstly, there are the gram-positive phi29-like phages, which form elongated tails through an elongated funnel-like tail tube structure [22–24]. Secondly, there are the gram-negative N4-like phages, which mainly rely on an elongated tail tube [13,25,26]. And thirdly, there are the gram-negative human gut viruses, whose tail length is increased by the tandem arrangement of multiple adaptor and muzzle proteins [27,76,77]. The elongated tail of A1002 appears to exhibit a greater degree of similarity to the first and second types, albeit with discernible differences, as A1002 forms a more complex double-layered tail tube. We hypothesize that this unique elongated tail structure of A1002 may have a dual function. Firstly, it may provide necessary spatial docking sites for multiple fiber dimers of A1002, supporting the anchoring and movement of its chain-like fibers. Secondly, it may assist in enhancing A1002’s capacity to penetrate *P. aeruginosa* extracellular structures, such as biofilms and capsules [30]. These structures are often strongly induced by environmental stresses, including antibiotics, phage attacks, oxidative stress, or host immunity [78]. It is well known that bacterial extracellular structures (such as biofilms and capsules) of gram-negative bacteria and the thick cell walls of gram-positive bacteria can act as physical barriers, hindering phage adsorption and infection on the host surface. Notably, among reported phages, P68-like phages that infect thick-walled Gram-positive bacteria [22] and the N4-like phage DEV, which can degrade the extracellular structures of *P. aeruginosa* [13], have independently evolved elongated tail tube structures. Although the morphologies of these tail tubes vary, they likely share similar functions in increasing phage tail length and helping overcome host physical barriers. Therefore, this feature of potentially enhancing host penetration capabilities via an elongated tail structure may represent a convergent evolution phenomenon across different phage lineages.

In summary, A1002 has acquired the three advantages: firstly, a more extensive search for host range through a unique nozzle complex, which is hypothesized to have originated from modular evolution and horizontal gene transfer; secondly, the dual-component dimer of its chain-like fibers, which is theorized to provide a structural basis for the highly efficient recognition of drug-resistant bacteria; and thirdly, the elongated, double-layered tail tube, which is predicted to offer an advantage in overcoming the extracellular barriers during *P. aeruginosa* infection. The identification of these distinctive structures offers a partial elucidation of the molecular mechanisms by which A1002 extensively infects various drug-resistant *P. aeruginosa* strains from a structural biology perspective. Moreover, it furnishes a novel molecular blueprint for the rational design of structure-based phage therapies.

## Materials and methods

### Production and purification of A1002

*P. aeruginosa* BP38-1 was cultured in TSB medium (17 g Tryptone, 3 g Soytone, 5 g sodium chloride, 2.5 g dipotassium hydrogen phosphate, and 2.5 g glucose per liter). Specifically, an overnight culture of *P. aeruginosa* BP38-1 was used to inoculate 5 mL of fresh TSB medium with 50 µL of the overnight culture, and the cells were grown at 37 °C with shaking at 220 rpm until the mid-logarithmic phase (approximately 4 h). Phage A1002 (suggest using an MOI of 1–10) was then co-incubated with an equal volume (300 µL each) of the log-phase host culture in a water bath at 37 °C for 10 min. A 200 µL aliquot of the phage‑bacterium mixture was transferred into a test tube containing 5 mL of TSB medium, and incubated at 37 °C with shaking at 220 rpm for 4 h. For bulk propagation, 15 mL of the phage culture obtained from the previous step was mixed with 15 mL of log‑phase host culture, and the mixture was incubated at 37 °C for 10 min. A 10-mL portion of this mixture was then inoculated into each of three Erlenmeyer flasks containing 300 mL of TSB medium (total approximately 1 L), and the flasks were incubated at 37 °C with shaking at 220 rpm for 4–6 h. When the culture became clear, cell debris was removed by centrifugation at 7,000 × *g* for 30 min at 8 °C. The resulting supernatant, containing the phage particles, was precipitated with 1 M NaCl and 10% PEG8000, and incubated at 4 °C for overnight in an ice-water bath. The phage precipitate was collected via centrifugation at 11,000 × *g* for 45 min and subsequently resuspended in PBS buffer. For further purification, the phage suspension was subjected to ultracentrifugation through a CsCl step gradient (1.7, 1.5, 1.4, and 1.3 g/mL) at 128,600 × *g* for 2 hours at 4 °C. The visible phage band was collected and dialyzed in PBS buffer at 4 °C overnight to remove CsCl. The A1002 particles were then evaluated using an electron microscope after negative staining. Finally, the mature phage preparations were stored in ice water for cryo-EM sample preparation.

### Data acquisition and image processing

Aliquots (3 µL) of the purified phage A1002 were deposited onto the Quantifoil R2/2 300-mesh Cu grids, which had been glow-discharged for 30 s. The grids were then loaded into an FEI Vitrobot maintained at 100% humidity and 8 °C, with a blot force of 3.5, and blotted for 4 s. Subsequently, the samples were vitrified by rapid plunging into liquid ethane and then transferred to liquid nitrogen for storage. Cryo-EM data were collected on an FEI Titan Krios G4 300 kV electron microscope, equipped with a Falcon 4i camera at a magnification of 96,000×, corresponding to a calibrated pixel size of 0.83 Å/pixel. Spherical aberration correction was applied to achieve a Cs value of approximately 2.7 mm. Each movie received an accumulated dose of approximately 32 e^−^/Å^2^. A total of 8,214 movie stacks, each comprising 32 frames, were recorded for mature A1002 particles. The multi-frame movies were drift-corrected using MotionCor2-1.4 [79], followed by CTF estimation with GCTF-1.06 [80] to determine defocus and astigmatism parameters. Approximately 200 representative particles from 40 micrographs were manually selected to generate templates for automated particle picking in RELION-3.14 [34]. Following automated selection and manual inspection, 23,000 particles (box size 600 × 600 pixels) were obtained from 5,776 micrographs and subjected to 2D classification into five classes. Two well-defined classes containing all 23,000 particles were used to generate an initial model. Subsequent 3D classification into five classes yielded three suitable classes for and Homogeneous Refinement, ultimately obtaining the structure of an icosahedral capsid at a resolution of 3.4 Å (EMDB ID: 83996).

### Asymmetric reconstruction and local reconstruction

We first determined the icosahedral capsid structure of the A1002 sample using RELION-3.14 [34]. We then imported the resulting particle orientation files into cryoSPARC-4.4.1 [35] by “Import Particles” function for icosahedral symmetry expansion. Subsequently, using UCSF ChimeraX 1.12 [81], we precisely localized the three-dimensional coordinates of the 5-fold vertex on the capsid density map at (*x*, *y*, *z*) = (282, 830, 498 Å) and re-centered the particle boxes to this vertex using the Volume Alignment tool in cryoSPARC [35]. Following this, duplicate particles were removed using a distance threshold of 40 Å. Local particles were then extracted with a box size of 400 × 400 pixels and subjected to 3D classification, which successfully separated the tail-less 5-fold capsid vertices (Class I) from the unique vertices harboring tail structures (Class II). Building on this, independent 3D reconstructions were performed for the two classes under distinct symmetry constraints. C5 symmetry and Homogeneous Refinement was applied to Class I, using 10,233 particles, yielding 2.8 and 3.2 Å resolution structures of the 5-fold vertex region (head penton) (EMDB ID: 80553, box size 400 × 400; EMDB ID: 82742, box size 600 × 600), while C6 symmetry and Homogeneous Refinement was applied to Class II, using 34,955 particles, resulting in a 3.1 Å reconstruction of the portal-nozzle complex (EMDB ID: 80605). Furthermore, in order to focus on the collar region surrounding the portal-nozzle complex, we applied a soft mask and re‑extracted 34,955 particles with a reduced box size of 512 × 512 pixels. We ultimately reconstructed a high-quality structure of the collar region at 3.1 Å resolution with C3 symmetry imposed (EMDB ID: 83847).

Additionally, in order to obtain the complete tail structure, we shifted the particle centers by 163 Å along the Z-axis and re-extracted the particles with a box size of 700 × 700 pixels. Following 2D classification, 14,932 particles were selected and subjected to local refinement with C6 symmetry imposed, yielding the overall tail structure at a resolution of 3.3 Å (EMDB ID: 82741). In this overall reconstruction, the resolutions of fiber dimers 1–7 and the tail tip structure remained relatively low. To address this, we performed focused reconstructions targeting these specific regions. The particles underwent C6 symmetry expansion, with their centers re-centered to the local fiber center at (*x*, *y*, *z*) = (327, 212, 88 Å). Subsequent using 88,970 particles and a box size of 160 × 160 pixels, we then performed local refinement under C1 symmetry with a soft mask, ultimately obtaining a local fiber map at a resolution of 3.8 Å (EMDB ID: 80643). In all refinement processes, no translational or rotational offset constraints were applied.

### Atomic model building and refinement

Based on the cryo-EM density map of the mature A1002 combined with AlphaFold3 predictions [33], the atomic models of the MCP gp50, CP gp49, portal protein gp52, adaptor protein gp47, collar protein gp33, nozzle-rope protein gp39, nozzle-base protein gp42, tail tube protein gp37, fiber protein gp41 and gp40 were built using modelAngelo software [53], followed by manual correction in WinCoot-1.1.20.1 software [82]. The needle protein gp29 were modeled using AlphaFold3 [33]. All models were refined with real-space refinement implemented in Phenix-1.21.2-5419 [83]. Interactions between capsomers and between tail fibers were analyzed using PDBePISA [84]. For each identified binding interface, we calculated the number of interface residues, interface area (Å^2^), number of salt bridges, number of hydrogen bonds, and number of non‑bonded contacts (S2 Table). We used the DALI server [54,55] with a Z-score threshold of approximately ≥7.0, and TM-align [70] with default parameters, retaining only hits with a TM-score approximately ≥0.55 and RMSD approximately ≤4.5 Å as significant matches. These criteria were consistently applied to the structural comparisons reported in MCPs, CPs, fiber dimers, portal, adaptor, and collar proteins among A1002 and other tailed phages. A summary of structural models for proteins of phage A1002 is shown in S3 Table.

## Supporting information

S1 Fig Cryo-EM image and Fourier shell correlation (FSC) curves of A1002. **(A)** A representative cryo-EM image of the A1002 particles. **(B)** Estimated structural resolutions of icosahedral capsid (EMDB ID: 83996), the head penton (EMDB ID: 80553/82742), portal-nozzle (EMDB ID: 80605), the overall tail (EMDB ID: 82741), the collar (EMDB ID: 83847), and the chain-like fiber at the tail tip (EMDB ID: 80643) in A1002. The FSC curves were calculated using the gold-standard procedure implemented in cryoSPARC. The resolution of the final map was estimated using the FSC = 0.143 criterion. FSC curves for all structural maps are presented for the masked and corrected half-maps, as the absence of masking and correction would cause noise surrounding the map to be included in the FSC calculation. **(C)** Local resolution maps of A1002. The data underlying this Figure can be found in S1–S6 Data.
(TIF)

S2 Fig Workflow for cryo-EM data processing and structure determination of A1002. The data underlying this Figure can be found in S1–S6 Data.
(TIF)

S3 Fig Cut-open view of the asymmetric structure and atomic models of 10 proteins from the head to the tail of A1002. All color codes are identical to those used in Fig 1A. The data underlying this Figure can be found in S1–S6 Data.
(TIF)

S4 Fig Structure of the MCP gp50. **(A)** Structural comparisons of the MCPs among phages A1002 gp50 (PDB ID: 44NY), E217 gp25 (PDB ID: 8FRS), R4C gp6 (PDB ID: 8GTA), Pa193 gp26 (PDB ID: 9B40), and T4 gp23* (PDB ID: 8GMO). Pairwise structural comparisons of A1002 gp50 with homologous proteins revealed the following results: E217 gp25 (RMSD = 4.37 Å, TM-score = 0.61), R4C gp6 (RMSD = 3.03 Å, TM-score = 0.61), Pa193 gp26 (RMSD = 4.29 Å, TM-score = 0.61), and T4 gp23 (RMSD = 4.05 Å, TM-score = 0.63). **(B)** Left: Superimposition of the nine MCPs, displayed in different colors. Right: Structural comparisons of MCPs from the hexon and the penton showing different conformations in A-domains. **(C)** Side views of ribbon models of a hexon and a penton (PDB ID: 26DE/44NY), with a monomer of each capsomer colored in orange-red.
(TIF)

S5 Fig Structural comparisons of the CPs of A1002 and other phages. **(A)** A1002 gp49 (PDB ID: 44NY). **(B)** KHP30 gp15 (PDB ID: 7DN2). **(C)** Pam1 gp6 (PDB ID: 7EEL). **(D)** BPP-1 Bbp16 (PDB ID: 3J4U). Pairwise structural comparisons of A1002 gp49 with its structural homologs yielded the following results: KHP30 gp15 (RMSD = 2.94 Å, TM-score = 0.66), Pam1 gp6 (RMSD = 2.64 Å, TM-score = 0.68), and BPP-1 Bbp16 (RMSD = 3.09 Å, TM-score = 0.56). All proteins are colored in rainbow, with the blue-to-red transition denoting the progression from the N-terminus to the C-terminus.
(TIF)

S6 Fig Interactions of the capsid capsomeres and cement proteins. **(A)** Top view of the interactions along a quasi-3-fold axis (EMDB ID: 82742), the data underlying this Figure can be found in S2 Data. The three hexon capsomeres and the CP trimer are colored orange, deep sky blue, lime green, and rose red, respectively (PDB ID: 26DE/44NY). **(B)** Top view of the CP. **(C)** Zoom-in view of the interaction between two adjacent CP monomers. A salt-bridge interaction is formed between residue K122 of one CP monomer (rose red) and residue E130 of the other monomer (light red). **(D)** Interaction between MCP monomers from two different capsomeres. Salt-bridge interactions between sidechain D114 of one capsomere and K60 of the adjacent capsomere, and between R116 of one capsomere and D100 of the adjacent capsomere. **(E)** Interaction of a CP monomer with two MCP monomers derived from two adjacent capsomeres. Hydrogen-bond interactions between residue T136 of CP and residue K339 of the MCP monomers, and between residue T136 of CP and residue N81 of MCP.
(TIF)

S7 Fig Structural comparisons of the portal proteins of phages A1002 gp52 (PDB ID: 26FB), DEV gp80 (PDB ID: 9BGM), N4 gp59 (PDB ID: 9LC1), Pam1 gp3 (PDB ID: 7EEP) and P-SCSP1u gp16 (PDB ID: 8I4M). Pairwise structural comparisons of A1002 gp52 with its structural homologs revealed significant similarities to DEV gp80 (RMSD = 5.12 Å, TM-score = 0.61), N4 gp59 (RMSD = 4.42 Å, TM-score = 0.72), Pam1 gp3 (RMSD = 4.92 Å, TM-score = 0.59), and P-SCSP1u gp16 (RMSD = 4.72 Å, TM-score = 0.60). All proteins are colored according to their domains.
(TIF)

S8 Fig Structural comparisons of the adaptor proteins of phages A1002 gp47 (PDB ID: 26FB), Carin-1 gp46 (PDB ID: 8CK1), SF6 gp7 (PDB ID: 7SP4), SU10 gp11 (PDB ID: 7Z47) and RcGTA Rcc01688 (PDB ID: 6TE9). Pairwise structural comparisons of A1002 gp47 revealed clear structural homologs, including Carin-1 gp46 (RMSD = 3.47 Å, TM-score = 0.66) and SU10 gp11 (RMSD = 2.88 Å, TM-score = 0.75), SF6 gp7 (RMSD = 3.57 Å, TM-score = 0.43) and RcGTA Rcc01688 (RMSD = 4.90 Å, TM-score = 0.44). All proteins are colored according to their domains.
(TIF)

S9 Fig Structure of the collar gp33 of A1002. **(A)** Structural comparisons of the collar proteins of phages A1002 (PDB ID: 46KE), Milano gp13 (PDB ID: 8FWC), DEV gp53 (PDB ID: 9COD), and GP4 gp77 (PDB ID: 8JOU). Pairwise structural comparisons of the A1002 collar gp33 with other phage proteins did not identify any significant structural homologs, including Milano gp13 (RMSD = 4.08 Å, TM-score = 0.40), DEV gp53 (RMSD = 3.30 Å, TM-score = 0.30), and GP4 gp77 (RMSD = 2.82 Å, TM-score = 0.35). All proteins are colored in rainbow, with the blue-to-red transition denoting the transition from the N-terminus to the C-terminus. **(B)** The interactional surface between 4 adaptor gp47 monomers and 5 collar gp33 monomers forming electrostatic complementary potential. Molecular surface electrostatic potentials are calculated using the Coulombic method implemented in ChimeraX software. The electrostatic potential scale is shown in the color bar.
(TIF)

S10 Fig Structural comparisons of the nozzle proteins of phages A1002 gp39 (PDB ID: 44NX/26GP) and gp42 (PDB ID: 26FB), SF6 gp8 (PDB ID: 7SG7), Pam1 gp8 (PDB ID: 7EEQ), VP1 gp16 (PDB ID: 8ZKK), E1004 gp25 (PDB ID: 9VVR) and Carin-1 gp44 (PDB ID: 8CK1). Pairwise structural comparisons of the A1002 nozzle-rope protein gp39 with various phage proteins did not identify any significant structural homologs, including SF6 gp8 (RMSD = 3.32 Å, TM-score = 0.23), Pam1 gp8 (RMSD = 3.51 Å, TM-score = 0.22), VP1 gp16 (RMSD = 3.44 Å, TM-score = 0.25), E1004 gp25 (RMSD = 3.64 Å, TM-score = 0.23), and Carin-1 gp44 (RMSD = 3.47 Å, TM-score = 0.23). In contrast, the A1002 nozzle-base protein gp42 showed significant structural similarities with the same set of proteins, including SF6 gp8 (RMSD = 4.42 Å, TM-score = 0.57), Pam1 gp8 (RMSD = 4.02 Å, TM-score = 0.54), VP1 gp16 (RMSD = 3.68 Å, TM-score = 0.62), E1004 gp25 (RMSD = 5.02 Å, TM-score = 0.59), and Carin-1 gp44 (RMSD = 4.37 Å, TM-score = 0.64), All proteins are colored according to their domains.
(TIF)

S11 Fig Structures of the tail tube of phages. **(A)** Structural comparisons of the tail tube of phage N4 gp54 (PDB ID: 9LC0), DEV gp75 (PDB ID: 9BGM), Andhra gp16 (PDB ID: 8EGR) and P68 gp18 (PDB ID: 6IAC). **(B)** Cut-open (left), side (middle) and zoomed-in (right) views of the electrostatic potential of portal-tail complex. Molecular surface electrostatic potentials are calculated using the Coulombic method implemented in ChimeraX software. The electrostatic potential scale is shown in the color bar.
(TIF)

S12 Fig Structural comparisons of the needle proteins of phages and pyocins. **(A)** A1002 gp29 modeled by AlphaFold3. **(B)** TP901 gp8 (PDB ID: 3EJC). **(C)** P2 gp6 (PDB ID: 3DA0). **(D)** Lambda gpJ (PDB ID: 8XCK). **(E)** R1 and R2 receptor-binding proteins (PDB ID: 6CL5 and 6CL6).
(TIF)

S1 Table Refinement and model statistics. (XLSX)

S2 Table Summary of all binding interfaces, interactions, and energetics in the A1002 capsid and fiber, as calculated by PISA. (XLSX)

S3 Table Summary of structural models for proteins of phage A1002. (XLSX)

S1 Data FSC data for the reconstruction of the A1002 phage icosahedral capsid structure. Data used to generate Figs 1 and S1–S3.
(XLSX)

S2 Data FSC data for the reconstruction of the A1002 phage head protein (box size 400 × 400 × 400). Data used to generate Figs 1 and S1–S3.
(XLSX)

S3 Data FSC data for the reconstruction of the A1002 phage portal-nozzle. Data used to generate Figs 1 and S1–S3.
(XLSX)

S4 Data FSC data for the reconstruction of the A1002 phage collar. Data used to generate Figs 1 and 2, as well as S1–S3 Figs.
(XLSX)

S5 Data FSC data for the reconstruction of the A1002 phage overall tail. Data used to generate Figs 1, 4, and 5, as well as S1–S3 Figs.
(XLSX)

S6 Data FSC data for the reconstruction of the A1002 phage local fiber. Data used to generate Figs 1, 4, and 5, as well as S1–S3 Figs.
(XLSX)

## Abbreviations

CP: cement protein
CRPA: carbapenem-resistant *P. aeruginosa*
FSC: Fourier shell correlation
MCP: major capsid protein
MDR: multidrug-resistant
PDR: pandrug-resistant
RMSD: root mean square deviation value
WHO: World Health Organization
XDR: extensively drug-resistant
